# *Salmonella* Characterization in Poultry Eggs Sold in Farms and Markets in Relation to Handling and Biosecurity Practices in Ogun State, Nigeria

**DOI:** 10.3390/antibiotics10070773

**Published:** 2021-06-24

**Authors:** Michael Agbaje, Patience Ayo-Ajayi, Olugbenga Kehinde, Ezekiel Omoshaba, Morenike Dipeolu, Folorunso O. Fasina

**Affiliations:** 1 Department of Veterinary Microbiology, College of Veterinary Medicine, Federal University of Agriculture, Abeokuta 110124, Nigeria; mikeagbaje@gmail.com (M.A.); omoshabaeo@funaab.edu.ng (E.O.); 2 Department of Veterinary and Pest Control Services, Federal Ministry of Agriculture and Rural Development, Abuja 900287, Nigeria; drtoyinayo@gmail.com; 3 Nigerian Field Epidemiology and Laboratory Training Programe, 50 Haille Selassie Str., Asokoro, Abuja 900287, Nigeria; 4 Department of Veterinary Public Health and Preventive Medicine, College of Veterinary Medicine, Federal University of Agriculture, Abeokuta 110124, Nigeria; gbengakehinde210@yahoo.com (O.K.); drmorenike@gmail.com (M.D.); 5 Emergency Centre for Transboundary Animal Diseases, Food and Agriculture Organization of the United Nations, Dar es Salaam 14111, Tanzania; 6 Department of Veterinary Tropical Diseases, Faculty of Veterinary Science, University of Pretoria, Onderstepoort 0110, South Africa

**Keywords:** antimicrobial resistance, biosecurity, egg, Nigeria, poultry, *Salmonella*

## Abstract

*Salmonella* remains one of the notable food-borne bacterial pathogens. It is associated with poultry and poultry products including eggs. This study investigated *Salmonella* distribution in eggshell and content, their antimicrobial resistance pattern, and the possible risk factors driving contamination in Ogun State, Nigeria. A total of 500 eggs (5 eggs pooled into one sample) were collected and culturally examined for the presence of Salmonella serovars. Isolates were further characterized biochemically using Microbact 20E (Oxoid) and Antimicrobial susceptibility determined by the Kirby-Bauer disk diffusion method. A total of 14 *Salmonella* isolates spread across 10 serovars were recovered from the 100 pooled egg samples; 10 (10%) from the market and 4 (4%) farms, 13(13%) eggshell, and 1(1%) egg content. All tested serovars were susceptible to ampicillin, chloramphenicol, florfenicol, and kanamycin. Resistance was mostly observed in sulfamethoxazole 8 (80%), followed by ciprofloxacin 5 (50%) and tetracycline 3 (30%). Sales of eggs in the market appear to be a strong factor encouraging contamination in addition to poor biosecurity and unhygienic handling of eggs on the farm.

## 1. Introduction

Poultry eggs provide a significant amount of animal protein in Nigeria and other developing Sub-Saharan nations since they are cheap, available, and have little or no limitation in acceptance across the socio-cultural and religious divide [1,2]. With a poultry population of approximately 180 million, Nigeria produces an average of 3.8 million eggs annually [3]. However, this important agricultural sector is burdened by infectious diseases including *Salmonella enterica* [4]. Recently a national survey reported *Salmonella* prevalence to be 43.6% among commercial poultry farms in Nigeria [5].

Salmonellosis is an important public health burden in most developing countries and constitutes a major food-borne pathogen in the developed world [6]. The non-typhoidal (NT) *Salmonella* species are largely self-limiting but serious consequences may result where infected individuals are immune-compromised or co-infected with malaria or human immunodeficiency virus (HIV) [7,8]. In the United States, about 1.4 million people are infected annually, with approximately 15,000 hospitalizations and 4000 deaths [9].

Traditionally, antimicrobials such as ampicillin, chloramphenicol, and trimethoprim-sulfamethoxazole were drugs of choice for the treatment of salmonellosis [10]. However, the emerging antimicrobial resistance trends in the last three decades have greatly diminished their efficacies against salmonellosis. These days, fluoroquinolones and third-generation cephalosporins are mostly preferred. Even at that, there have been increasingly reported cases of resistance to fluoroquinolones and third-generation cephalosporins [11,12].

Non-typhoidal *Salmonella* species, like other bacterial pathogens, can acquire resistance to antimicrobial agents, thereby enhancing their pathogenicity, virulence, and impact on the infected human population [13]. While bacterial resistance is commonly acquired through mechanisms such as mutations and/or horizontal plasmid transfer, the transmission of resistant NT *Salmonella* to humans is mostly associated with contact with infected animals and consumption of contaminated foods, especially foods of animal origin such as poultry, fish, eggs, beef, and dairy products [12,14].

Contamination in the food chain by antimicrobial-resistant bacteria and resistance elements such as genes and plasmids are traceable to the intense use of antimicrobials in livestock production and the selective pressure that develop against bacterial organisms such as NT *Salmonella* sp, leading to the emergence of resistance against them [15,16].

In Nigeria, NT *Salmonella* has been mostly characterized in the poultry sector from feces, dust, environment, and poultry meat [5,12,17,18]. However, information on the drivers of egg contamination and transmission is scanty due to the lack of a coordinated national surveillance program. Also, increasing reports of multi-drug resistance to antimicrobial agents from *Salmonella* strains isolated from eggshells and contents [19,20] signals a need for continuous collection of data in the poultry sector to aid relevant authorities in decision making and response in Nigeria. Consequent to the above, this study seeks to establish: a) a baseline survey of *Salmonella* occurrence in poultry eggs, ii) determine the circulating *Salmonella* serovars and their antimicrobial resistance profile, and iii) determine possible risk factors that may be driving *Salmonella* contamination of eggs.

## 2. Results

### 2.1. Prevalence and Diversity of Salmonella Isolates on Shells and in Contents of Eggs

Of the 100 samples pooled from 500 eggs, 14 (14%) from markets (*n =* 10), and farms (*n =* 4) were positive for *Salmonella* spread across shell (13/14, 92.9%) and content (1/14; 7.1%) (Table 1). *Salmonella* in eggshell was significantly higher (*p* < 0.05) than that of egg content.

Ten (10) *Salmonella* isolates were obtained from 40 samples (i.e., 40 × 5 = 200) eggs sold in the markets while the remaining four (4) were from 60 samples (i.e., 60 × 5 = 300) eggs obtained from the farm. The most isolates were from Egba (*n =* 7) followed by Yewa (*n =* 4), Ijebu (*n =* 2) and Remo (*n =* 1). All *Salmonella* isolates were obtained from eggshell except one (S. Alachua) from the Remo zone which was obtained from egg contents (Table 1). In this study, 10 different *Salmonella* serovars were identified from 14 *Salmonella* isolates from eggs predominantly sold in the market and included: Agama, Durham, Bradford, Derby, and Kentucky. Only serovars Kentucky was common in samples from the market and farms. Other serovars identified in the farm-sourced eggs include Kingston, Colorado, Lattenkamp, Carno, and Alachua (Table 1).

### 2.2. Salmonella Serovar Resistance to Antimicrobials and Their Resistance Patterns

The antibiotic resistance profile of fourteen *Salmonella* serovars was subjected to 11 commonly used antimicrobial agents (Table 2). All tested serovars were susceptible to ampicillin, chloramphenicol, florfenicol, and kanamycin. Resistance was most predominantly shown to sulfamethoxazole 8 (80%), followed by ciprofloxacin 5 (50%) and tetracycline 3 (30%). Gentamicin, nalidixic acid, and streptomycin showed equal resistance 2 (20%) with the least resistance observed in trimethoprim 1 (10%) (Table 2). The most resistant *Salmonella* serovar to antimicrobials was *S*. Kentucky (*n =* 6), followed by S. Carno (*n =* 4) and S. Derby (*n =* 3). There was no reissuance shown to any of the antimicrobials by S. Colorado (Table 2).

Of the 14 positive *Salmonella* isolates spread across 10 serovars, five were resistant to two or more antimicrobials and included Kentucky, Bradford, Derby, Carno, and Kingston while four including Agama, Lattenkamp, Durham, and Alachua showed resistance to single antimicrobials (Table 2). For the 7 *Salmonella* isolates tested from the Egba zone, only one multi-resistance pattern (SMX-GEN-TET-STR-CIP-NAL) was observed in comparison to two patterns each from Yewa (SMX-CIP; SMX-TET-CIP) and Ijebu (CIP-NAL; SMX-GEN-STR-TMP) with four and two isolates respectively (Table 3).

### 2.3. Biosecurity Practices in the Production and Handling of Eggs

The questionnaire results indicated a predominant cage system (73.3%) operations, compared to the deep litter system (26.7%). Eighty percent (48/60) of the farms were less than 500 m away from other farms and the tendency for farms to be visited by wild birds. Twenty percent of the farms included antibiotics in their poultry feeds routinely.

Wide biosecurity concerns exist across most farms with only about 21.6% (13/60) of farm operations involveing personal protective equipment (PPEs) where necessary. About half of the responding farms shared tools with other farms, thereby encouraging pathogen transfer. All respondents (100%) did not clean their eggs in any form before selling (Table 4). A fifth (12/60) of the respondents include antibiotics in feed as growth promoters or prophylactics while 4168.3% (41/60) of the respondents allow in-farms sales of eggs (Table 4).

## 3. Discussion

In this study, a prevalence of 14% non-typhoidal *Salmonella* was detected from pooled egg samples. To the best of our knowledge, this study provides the first detailed comparison of *Salmonella* serovars profile sold on-farm and in the open market. Our results corroborate *Salmonella* presence in eggs in Nigeria as previously reported [21]. A previous national study reported a 24.5% prevalence of NT *Salmonella* in poultry environments in Ogun State (1). The differences in the prevalence of the two studies may be attributed to the sample types investigated. While the national study employed a matrix of five samples (dust, litter, feces, feed, and water) from poultry environments, the current study focused on pooled poultry egg samples.

The occurrence of *Salmonella* in eggs from markets and egg shell was significantly higher. Contamination of eggs may occur during packing, grading, transporting and sales in the market, as multiple buyers visually inspect, touch, and select eggs during sales in the study area [22]. In the present study, unhygienic egg handling practices were common in all farms and markets involved. Also, all farms involved in the questionnaire survey have no egg sanitation programs in place. Data from this study further highlight the potentials continuous relevance of poultry eggs as an important transmission reservoir of *Salmonella* in humans. Thirteen out of the 14 *Salmonella* isolates identified in this study were found on the eggshells and may suggest fecal, environmental, or handling contamination [23]. Only one *Salmonella* serovar (*S*. Alachua) was detected in egg content, but, the route of contamination was not investigated. *S*. Alachua was recently reported from fecal samples in the Northern part of Nigeria [24]. Further study will be required to determine if *S*. Alachua was an accidental finding in the egg content, vertically transmissible, or can penetrate eggshells into the contents.

The fourteen *Salmonella* isolates identified in this study were spread across 10 serovars, which depicts high serovar diversity. Studies across Nigeria have reported similar observations [5,12,24]. Plausible reasons for this findings are the indiscriminate importation of poultry birds and eggs with no coordinated national screening and control program in place for salmonellosis. While *S*. Agama (*n =* 3) was the most occurring in this study, all three isolates were from the same market and zone. It is then possible that all three are clonally related, although clonal relatedness was not explored in this study. Two *S*. Kentucky serovars were identified, each from a different zone. Fagbamila et al. [5] reported *S*. Kentucky in 11 states out of the 12 that were sampled in Nigeria. Other studies have similarly reported *S*. Kentucky across Nigeria, thereby suggesting this serovar as widely circulating in Nigeria [17,24,25,26]. *S*. Kentucky has a worldwide distribution and was previously thought to be endemic in Africa with public health significance [27,28].

Notably in this study, *Salmonella* serovars commonly associated with foodborne infections (*S*. Enteritidis and *S*. Typhimurium) [29] were absent. The *S*. Gallinarum vaccine commonly used in Nigeria may protect against other group D-strains such as *S*. Enteritidis [5,30]. In addition, Fagbamila et al., [5] and Useh et al., [26], have suggested that these two serovars likely play minor roles in the Nigerian poultry sector. Put together, serovar diversity may be attributable to a number of reasons but not limited to poor sanitary and biosecurity conditions, indiscriminate importation of poultry chickens and eggs without adequate screening for *Salmonella,* and lack of focused national *Salmonella* surveillance and control program.

The abuse and misuse of antimicrobial agents in the poultry sector have been linked to increased resistance to antimicrobials [31]. In this study, antimicrobial resistance to *Salmonella* was highest in sulfamethoxazole (SMX), followed by ciprofloxacin (CIP) and tetracycline (TET) respectively. A previous study on NT *Salmonella* occurrence in freshly dressed poultry meat in northern Nigeria reported a high *Salmonella* resistance pattern to SMX, CIP, and TET [12]. This emerging resistance pattern is corroborated by studies on veterinary students’ ranked perception of abused antimicrobials in Africa in which sulphonamides and tetracycline were in the uppermost three ranked antimicrobials [32,33]. However, in contrast to our study, earlier investigations in Zimbabwe by Makaya et al. [34] and Adesiyun et al. [35] in the Caribbean region reported no resistance to SMX. It is then possible that increased use and misuse of SMX in the Nigerian poultry sector may be a driving factor in the resistance observed in SMX. In addition, resistance to ciprofloxacin raises concern since this is the drug of choice in the treatment of human invasive salmonellosis. Resistance to tetracycline is not surprising considering its extensive prophylactic usage and as additives in feed and water to enhance performance in Nigeria [16]. It may then be inferred that the frequency of *Salmonella* spp. resistance to these antimicrobials reflects their intense application in the poultry sector in Nigeria. The observed lack of stringent control on the availability and non-prescription use of antibiotics in poultry practice in the study area is concerning. In Nigeria, over the counter availability of most antimicrobials in local drug stores makes the control of antibiotic usage cumbersome [18].

In this study, data from the questionnaire indicated about 90% of the farms in the study area are accessed by wild birds and rodents. Similar to our results, a study involving three Caribbean countries also reported rodents in 90% of contaminated farms [36]. Investigations in Australia have demonstrated the role of environmental vectors in the epidemiology of *Salmonella* in farm settlements [37,38]. High and unchecked rodent populations have been associated with increased *Salmonella* shedding in the environment [39] and are the most effective in the spread of *Salmonella* pathogen around farms [40]. It is then imperative to initiate robust vector prevention programs in farmhouses which may include secured access doors and windows, sealing of holes, repairs of torn wire net, and the use of baits to help control contamination of farmhouses [41].

Furthermore, our results revealed certain practices which may encourage *Salmonella* occurrence and/or persistence in farms. Poor adherence to strict biosecurity measures on farms. The use of protective clothing as a barrier to infectious agents was unpopular among the majority of the farmers and may contribute to increased chances of contamination. Also, certain high-risk cross-contamination practices such as unhygienic picking of eggs with bare hands, sharing of tools with nearby farms (mostly <500 m away), and sales of eggs on the farm were observed. These practices all increase the risk of contamination and transfer of pathogens [24,38,42].

The findings in this study are subject to at least two limitations. First, the pooling of samples ultimately reduced the sample size. While investigating individual egg samples will have provided more detailed data, pooled samples are considered more effective for the successful detection of *Salmonella* in the context of this study [43]. Second, was our inability to match individual samples collected with corresponding husbandry and biosecurity questionnaires in the data analyses. Considering that the positivity rate of *Salmonella* was 14%, it was considered that analyzing data as per the region will be more informative than individual sample-farm analysis.

## 4. Materials and Methods

### 4.1. Study Location

This observational study was conducted across poultry farm settlements and markets in Ogun State Nigeria. Ogun state is comprised of four socio-cultural zones (Egba, Ijebu, Yewa, and Remo) spread across 20 Local Government areas. Ogun State covers an area of 16,762 square kilometers and stands at an elevation of 169 feet with a population of 4,054,272 [44]. Ogun State occupies latitude 6.2–7.8° N and longitude 3.0–5.0° E. A stratified probability random sampling design was adopted for this study such that poultry farms and markets from the four zones of Ogun state were evenly represented in the final sample.

### 4.2. Sample Collection

Egg samples from poultry farms and markets were used for *Salmonella* determination. A total of five hundred (500) eggs were collected representing 125 eggs per each of the four zones. From each zone, 75 eggs were obtained from 3 poultry farms and 50 eggs from 2 markets. Samples were analyzed in pools of 5 making a total of 25 sample units per zone (15 from farms and 10 from markets). Samples were collected into sterile bags using sterile nylon gloves and transported to the laboratory at 37 °C.

### 4.3. Isolation of Salmonella

The egg surface was disinfected with 70% alcohol and alcohol residue removed by flaming. A sterile thumb forceps was used to aseptically separate the shell from the interior content. To pre-enrich, 5 mL of homogenized egg content was dispensed and thoroughly mixed with 45 mL of buffered peptone water (BPW, Oxoid CM1049) and incubated at 37 °C for 18 h.

Following pre-enrichment, 1 mL of pre-enriched broth was aseptically transferred into 9 mL of sterile Mueller-Kaufmann Tetrathionate novobiocin selective enrichment broth (MKTTn, Oxoid CM1048) and sufficiently homogenized. Similarly, 0.1 mL of the pre-enriched broth culture was dispensed into 9.9 mL of sterile modified semi-solid Rappaport Vassiliadis (MSRV) selective enrichment broth (MSRV, Oxoid CM0910) supplemented with novobiocin (Oxoid SR0161). Inoculated MKTTn and MRSV broth were incubated at 37 °C and 41.5 °C, respectively, for 24 h.

Following incubation, a loopful of observable bacterial growth was each taken from the MRVS and MKTTn broth cultures and streaked simultaneously on the surfaces of xylose lysine desoxycholate (XLD) and Mac-Conkey (MAC) agar plates. The inoculated plates were incubated at 37 °C for 18–24 h and then examined for bacterial growth. Bacterial colonies consistent with *Salmonella* growth on XLD agar (light red colonies, some with black centers) and MAC agar (pale/colorless translucent colonies)) were selected for further biochemical and serological characterization.

### 4.4. Biochemical Identification of Salmonella

Suspected colonies were subjected to catalase and oxidase tests. For further identification different biochemical tests were carried out using MICROBACT TM GNB 24E KIT (OXOID) for Gram-negative bacteria and the result was interpreted using the computer software package Oxoid Microbact^®^ 2000 (version 2.03).

### 4.5. Antibiotic Sensitivity Testing

The isolates were subjected to an antibiotic sensitivity test according to the Bauer-Kirby technique to evaluate the antimicrobial susceptibility pattern. Eleven (11) antibiotics commonly used in the study area were used namely; ampicillin (AMP, 10 μg), chloramphenicol (CHL, 30 μg), ciprofloxacin (CIP, 5 μg), florfenicol (FFN, 30 μg), gentamicin (GEN, 30 μg), kanamycin (KAN, 30 μg), nalidixic acid (NAL, 30 μg), streptomycin (STR, 10 μg), sulfamethoxazole (SMX, 25 μg), tetracycline (TET, 25 μg), trimethoprim (TMP, 5 μg).

### 4.6. Procedure

Briefly, the turbidity of test *Salmonella* isolates grown overnight in peptone water broth was adjusted to an equivalent of 0.5 McFarland concentration (approximately 1 × 10^8^ cfu/mL). *Salmonella* broth culture was then evenly applied to cover the entire surface of freshly prepared Mueller Hinton agar (MHA) plate using a sterile spreader. Antimicrobial disks were carefully and firmly placed on the MHA surface at equidistance and incubated at 35 ± 2 °C for 18 h. The diameter of the zone of inhibition around each disk was measured and the result was interpreted according to Clinical and Laboratory Standards Institute (CLSI) recommendations. Strains displaying intermediate resistance were regarded as resistant.

### 4.7. Serotyping of Salmonella

Presumptive *Salmonella* isolates were serotyped by agglutination tests with specific O and H antisera and classified according to the Kauffman-White scheme as previously described [45] at the Istituto Zooprofilattico Sperimentale delle Venezie, National and OIE Reference Laboratory for Salmonellosis, Legnaro, Italy.

### 4.8. Determination of Biosecurity Practices on Poultry Farms

A test questionnaire was distributed among 15 farms not included in the final data collection to determine the clarity of the questionnaire. Based on the feedback from the respondents, adjustments were made to the final questionnaire.

Sixty (60) well-structured questionnaires were administered to farmers and farms spread across the four zones of Ogun State to evaluate the husbandry practices and adherence to biosecurity measures on farms. Fifteen (15) questionnaires were distributed per zone and included farms from which egg samples were previously taken for *Salmonella* detection. Farmers were informed of their rights to discontinue participation at any stage of the project and anonymity and confidentiality of data were stressed.

### 4.9. Data Management and Statistical Analysis

Data collation and management were computed using Microsoft Excel. The responses to the questionnaire were presented in percentages; descriptive statistics were used to describe the prevalence analysis. The Pearson’s Chi-square values and *p*-values for proportions were determined for the data generated using Statistical Package for Social Sciences (SPSS) software, version 20.

## 5. Conclusions

This study demonstrated the presence of diverse NT *Salmonella* serovars in eggs with potential antimicrobial-resistant traits. Sales of eggs in the market seem to promote the risk of *Salmonella* contamination as well as other unhygienic biosecurity practices on the farm.

## Figures and Tables

**Table 1:** Zonal distribution of *Salmonella* serovars in eggs according to sources of samples and types.

Zone	Sources of Isolates				Identified Serovars	
	Market (%)	Farm (%)	Shell (%)	Contents (%)	Market (*n*)	Farm (*n*)
Egba (*n* = 25)	5/10 (50)	2/15 (13.3)	7/25 (28)	0	Agama (3), Colorado (1), Lattenkamp (1)	Kingston (1), Kentucky (1)
Yewa (*n* = 25)	4/10 (40)	0/15 (0)	4/25 (16)	0	Durham (2), Bradford (1), Derby (1)	-
Ijebu (*n* = 25)	1/10 (10)	1/15 (6.7)	2/25 (8)	0	Kentucky (1)	Carno (1)
Remo (*n* = 25)	0/10 (0)	1/15 (6.7)	0	1/25 (4)	-	Alachua (1)
Total	10/40 (25) *	4/60 (6.7)	13/100 (13) *	1/100 (1)		

* *p*-value is significant at <0.0001. Between markets and farms, Χ^2^ = 39.7, *p*-value < 0.0001; and between shell and contents, Χ^2^ = 63.9, *p*-value < 0.0001.

**Table 2:** Frequency resistance of *Salmonella* isolates by serovars.

Serotypes	No of Isolates Tested	No (%) of Resistant Isolates	No (%) of Resistant to Antimicrobials											Antmicr Type Resist (*n*)
			AMP	CHL	CIP	FFN	GEN	KAN	NAL	STR	SMX	TET	TMP	
Agama	3	1 (33.30)	-	-	-	-	-	-	-	-	1	-	-	1
Alachua	1	1 (100)	-	-	1	-	-	-	-	-	-	-	-	1
Bradford	1	1 (100)	-	-	1	-	-	-	-	-	1	-	-	2
Carno	1	1 (100)	-	-	-	-	1	-	-	1	1	-	1	4
Colorado	1	0 (0)	-	-	-	-	-	-	-	-	-	-	-	
Derby	1	1 (100)	-	-	1	-	-	-	-	-	1	1	-	3
Durham	2	1 (50)	-	-	-	-	-	-	-	-	1	-	-	1
Kentucky	2	2 (100)	-	-	2	-	1	-	2	1	1	1	-	6
Kingston	1	1 (100)	-	-	-	-	-	-	-	-	1	1	-	2
Lattenkamp	1	1 (100)	-	-	-	-	-	-	-	-	1	-	-	1
Total	14	10 (71.4) *	0	0	5 (50)	0	2 (20)	0	2 (20)	2 (20)	8 (80)	3 (30)	1 (10)	

AMP: ampicillin; CHL: chloramphenicol; CIP: ciprofloxacin; FFN: florfenicol; GEN: gentamicin; KAN: kanamycin; NAL: nalidixic acid; STR: streptomycin; SMX: sulfamethoxazole; TET: tetracycline; TMP: trimethoprim. * Significant proportion of the tested isolates were resistant to selected antimicrobials (Χ^2^ = 4.9, *p*-value = 0.03). Antimic Type Resist = cumulative number of antimicrobial resistant to.

**Table 3:** Antimicrobial resistance patterns of *Salmonella* isolates from eggs in Ogun State, Nigeria.

Zones	No. (%) of Isolates	*Salmonella* Serovars (n)	Resistance Pattern
Egba	7 (50)	Agama (2), Lattenkamp (1), Kingston (1)	SMX (4)
		Kentucky (1)	SMX-GEN-TET-STR-CIP-NAL (1)
			
Yewa	4 (28.6)	Durham (1)	SMX (1)
		Bradford (1)	SMX-CIP (1)
		Derby (1)	SMX-TET-CIP (1)
			
Ijebu	2 (14.3)	Kentucky (1)	CIP-NAL (1)
		Carno (1)	SMX-GEN-STR-TMP (1)
			
Remo	1 (7.1)	Alachua (1)	CIP (1)

Note: All antimicrobials tested were fully sensitive to two S. Agama isolates in Egba and one S. Durham isolate in Yewa zones. AMP: ampicillin; CHL: chloramphenicol; CIP: ciprofloxacin; FFN: florfenicol; GEN: gentamicin; KAN: kanamycin; NAL: nalidixic acid; STR: streptomycin; SMX: sulfamethoxazole; TET: tetracycline; TMP: trimethoprim.

**Table 4:** Husbandry and biosecurity practices in the study area.

Items	Response	Frequency	Percentage	Χ^2^ (*p*-Value)
Husbandry system	Cage	44	73.3	25.9 (<0.0001)
	Deep litter	16	26.6	
Presence of other farms <500 m away	Yes	48	80.0	42.8 (<0.0001)
	No	12	20.0	
Presence of wild birds and rodents around the farm	Yes	53	88.0	68.7 (<0.0001)
	No	7	12.0	
				
Sanitation				
Wearing protective clothing	Yes	13	21.6	38.3 (<0.0001)
	No	47	78.3	
Sharing of tools with other farms	Yes	32	53.3	0.52 (0.47)
	No	28	46.6	
Cleaning of eggs before sale	Yes	0	0.0	119.0 (<0.0001)
	No	60	100.0	
Inclusion of antibiotics in feed	Yes	12	20.0	42.8 (<0.0001)
	No	48	80.0	
**Traffic control**	Farm premises	41	68.3	16.0 (0.0001)
Point of sale of eggs	Off-farm premises	19	31.6	

## Data Availability

Data sets used during the study are available from the corresponding author on reasonable request.
